# Cocaine Inebriety

**Published:** 1887-09

**Authors:** 


Cocaine Inebriety.—Dr. T. D. Crothers concludes a paper in his *Quarterly Journal of Inebriety* as follows:

"The use of cocaine to excess in persons who have never used alcoholic or other narcotic drugs before, is very rare. Among inebriates and drug maniacs, cocaine inebriety is no doubt increasing. Its peculiar dangerous effects on the body will prevent its general use as an intoxicant to any great extent. It acts more rapidly than opium, but its effects pass off more quickly. Its first effect is more exhilarant than alcohol, but it is uncertain and variable. This stimulant action develops mania, followed by narcotism and melancholia. When given in cases of melancholia in large doses, it changes the case to mania, then finally relapses, bringing back the case to melancholia again. As an intoxicant it is more dangerous than alcohol or opium. As a form of inebriety it is more difficult to treat, requiring a longer time to break up, because of the physical complications. It cannot be used as a substitute for any other narcotic, or as an antidote or remedy."—*Practice.*

